# Effect of video‐based intervention on the sun protective beliefs and behaviours of student athletes

**DOI:** 10.1002/ski2.318

**Published:** 2023-12-01

**Authors:** Austin N. Johnson, Peter W. Barnes, Matthew P. Dizon, Susan M. Swetter, Kristin M. Nord, Hayley W. Leatham

**Affiliations:** ^1^ Department of Dermatology Stanford University School of Medicine Stanford California USA; ^2^ Health Services Research and Development VA Palo Alto Health Care System Menlo Park California USA; ^3^ Dermatology Service VA Palo Alto Health Care System Palo Alto California USA

## Abstract

Prior work has demonstrated that a novel programme involving dermatologist‐led, team‐based education of student athletes (SAs), coaches, and athletic trainers termed Stanford University Network for Sun Protection, Outreach, Research, and Teamwork (SUNSPORT) improved photoprotective behaviours in National Collegiate Athletic Association (NCAA) SAs. Our current study investigated the use of an alternative, video‐based form of SUNSPORT at Cal and UCLA. We demonstrate a trend for increasing sunscreen use amongst SAs with a more feasible programme.

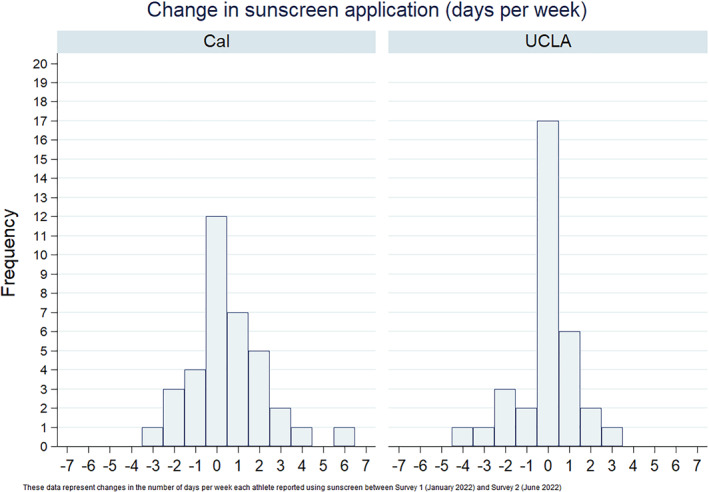

Dear Editor,

Excessive ultraviolet radiation (UVR) exposure early in life is a well‐established risk factor for skin cancer development.^1^ Despite evidence that regular sunscreen use reduces skin cancer incidence, adolescents and young adults infrequently engage in photoprotective behaviours.^2^ Low sunscreen use persists in high‐risk populations, including outdoor student athletes (SAs) who are repeatedly exposed to intense UVR during practice and competition.^3^, ^4^ In 2012, a novel programme involving dermatologist‐led, team‐based education of SAs, coaches, and athletic trainers called the Stanford University Network for Sun Protection, Outreach, Research, and Teamwork (SUNSPORT) improved photoprotective behaviours in National Collegiate Athletic Association (NCAA) SAs.^5^ Our current study aimed to evaluate an alternative, more feasible form of SUNSPORT utilising a brief educational video in place of the original intensive face to face educational sessions.

Following Stanford University IRB approval, baseline surveys assessing UVR exposure and sun‐protection beliefs and behaviours were distributed to outdoor spring season SAs at University of California, Los Angeles (UCLA) and University of California, Berkeley (Cal). Both sites were then provided sunscreen provisions for SA locker rooms, training sites, and travel. After 6 weeks, one school (Cal) was randomly selected to receive the video intervention, a 2‐min film which informed about UVR exposure, modelled sun‐protective practices, and included personal experiences of SAs with melanoma. After 20 weeks, a follow‐up survey was distributed to participants at both schools.

Overall, 192 SAs completed the initial survey (response rate 22.8%), with 36 Cal and 33 UCLA SAs completing both the baseline and post‐intervention surveys for an overall response rate of 8.2%. Of these SAs, 78% were female (54), with 81% aged 18–21 (56). The most common self‐reported Fitzpatrick skin types were II (35%; 24) and III (32%; 22), and the majority reported brown (55%; 38) or blond (36%; 25) hair. The dominant eye colour was brown (38%; 26). The SA cohort comprised 11 primary competitive sports, with 28% (19) being track and field SAs and 19% (13) competing in crew. There were also ten (14%) swimmers and seven (10%) beach volleyball players. These characteristics did not differ significantly between schools (*p* > 0.05 using Fisher's exact test). Baseline sunscreen use was similar at both sites, with 53% (19) of Cal SAs and 52% (17) of UCLA SAs reporting sunscreen application at least 4 days per week.

To gauge the effectiveness of the intervention, we compared the change in number of days per week SAs reported using sunscreen from baseline to follow‐up at the two schools using a two‐sample *t*‐test. Post‐intervention, Cal SAs reported a greater mean change in weekly sunscreen use (0.56 days vs. −0.06 days, *p* = 0.12). The change per SA from baseline at each school is shown in Figure 1. At follow‐up, 22% (8) of Cal SAs reported fewer weekly applications, 33% (12) maintained the same weekly applications, and 44% (16) increased applications after the intervention. In comparison, 21% (7) of UCLA SAs reported fewer applications, 52% (17) the same number of applications, and 27% (9) more applications.

**FIGURE 1:**
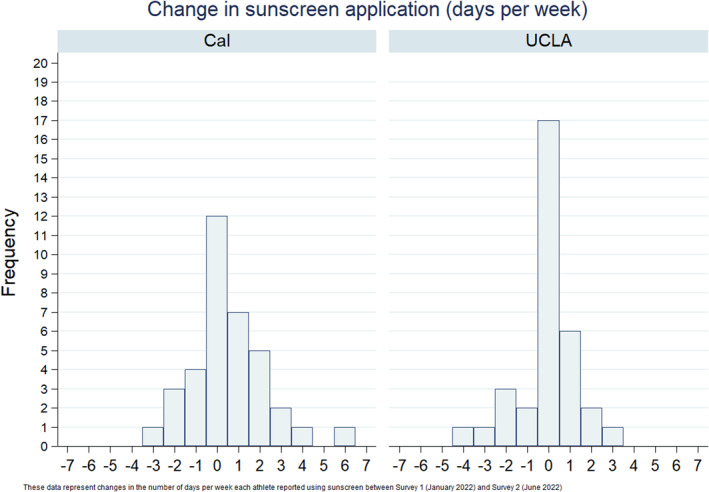
Post‐Intervention Change in Sunscreen Application Among student athlete (SA) (SA) Cohorts. The changes in the number of days of sunscreen application per week each SA reported between the pre‐ and post‐intervention surveys (January 2022 and June 2022, respectively).

Our previous SUNSPORT efforts involved direct interaction with SAs, coaches and athletic trainers and demonstrated increased sun‐protective behaviours in Stanford SAs through dermatologist‐led, 1‐on‐1 and team‐based education.^5^ The current study highlights the merits of a more reproducible SUNSPORT programme for SAs at other universities using a short video intervention and enhanced sunscreen access, suggesting that SUNSPORT (and similar programs across the NCAA) can increase SA sun‐protective behaviours with minimal time investment. Our findings also reinforce that availability of sunscreen and usage reminders can improve sun‐protective practices in SAs,^5^ highlighting the importance of these core objectives in any future educational programs. Although the difference in change in sunscreen use was not statistically significant, the positive trend was evident, and a significant effect may have been detected in a larger sample. Additionally, sunscreen use at both schools was higher at baseline compared to previously reported baseline sunscreen usage at Stanford, which may have limited the magnitude of the effect.^5^ Because participation was voluntary, our study is limited by a relatively low response rate, which may limit generalisability and introduce selection bias. Despite these limitations, our findings support the potential of this alternative form of SUNSPORT to increase sunscreen use without the demands of in‐person, dermatologist‐led education. Further investigation of similar educational outreach to improve sun‐protective practices among NCAA and other SAs is warranted.

## CONFLICT OF INTEREST STATEMENT

None to declare.

## AUTHOR CONTRIBUTIONS

**Austin N. Johnson**: Conceptualization (equal); Formal analysis (equal); Writing – original draft (equal); Writing – review & editing (equal). **Peter W. Barnes**: Conceptualization (equal); Data curation (equal); Formal analysis (equal); Investigation (equal); Methodology (equal); Writing – original draft (equal); Writing – review & editing (equal). **Matthew P. Dizon**: Methodology (equal); Writing – original draft (equal); Writing – review & editing (equal). **Susan M. Swetter**: Conceptualization (equal); Data curation (equal); Formal analysis (equal); Funding acquisition (equal); Investigation (equal); Methodology (equal); Project administration (equal); Supervision (equal); Validation (equal); Visualization (equal); Writing – original draft (equal); Writing – review & editing (equal). **Kristin M. Nord**: Conceptualization (equal); Data curation (equal); Formal analysis (equal); Investigation (equal); Methodology (equal); Project administration (equal); Resources (equal); Supervision (equal); Validation (equal); Visualization (equal); Writing – original draft (equal); Writing – review & editing (equal). **Hayley W. Leatham**: Conceptualization (equal); Data curation (equal); Formal analysis (equal); Investigation (equal); Methodology (equal); Project administration (equal); Validation (equal); Visualization (equal); Writing – original draft (equal); Writing – review & editing (equal).

## FUNDING INFORMATION

Mary E. Brenneisen Fund at Stanford Medicine.

## ETHICS STATEMENT

IRB Approval via Stanford: eProtocol #55906. Approved 6/11/2021.

## Data Availability

The data that support the findings of this study are available from the corresponding author upon reasonable request.
